# Healthy Lifestyle Is Associated with Reduced Mortality in Patients with Non-Alcoholic Fatty Liver Disease

**DOI:** 10.3390/nu14183785

**Published:** 2022-09-14

**Authors:** Chengxiao Yu, Jiaxin Gao, Xinyuan Ge, Xiao Wang, Yuqing Ding, Ting Tian, Xin Xu, Wen Guo, Quanrongzi Wang, Zijun Ge, Tao Jiang, Qun Zhang, Ci Song

**Affiliations:** 1 Department of Epidemiology, China International Cooperation Center on Environment and Human Health, Center for Global Health, School of Public Health, Nanjing Medical University, Nanjing 211166, China; 2 The Affiliated Suzhou Hospital of Nanjing Medical University, Suzhou Municipal Hospital, Gusu School, Nanjing Medical University, Suzhou 215008, China; 3 Department of Infectious Disease, The First Affiliated Hospital of Nanjing Medical University, Nanjing 210029, China; 4 Health Management Center, The First Affiliated Hospital of Nanjing Medical University, Nanjing 210029, China; 5 Department of Radiology, The First Affiliated Hospital of Nanjing Medical University, Nanjing 210029, China; 6 Office of Infection Management, The First Affiliated Hospital of Nanjing Medical University, Nanjing 210029, China; 7 Research Units of Cohort Study on Cardiovascular Diseases and Cancers, Chinese Academy of Medical Sciences, Beijing 100000, China

**Keywords:** NAFLD, mortality, lifestyle, cohort study, prevention

## Abstract

Background and Aims: It is unclear whether a healthy lifestyle impacts mortality in the presence of non-alcoholic fatty liver disease (NAFLD). The present study aimed to examine the joint association of several modifiable lifestyle factors with mortality risk for NAFLD patients. Methods: We collected lifestyle behavior data form the National Health and Nutrition Examination Survey (NHANES) III from 1988 to 1994 and follow-up data form NHANES III-linked mortality data through 2015. We estimated joint association between four healthy lifestyle factors (non-smoking, non-drinking, regular physical activity, a healthy diet) after NAFLD diagnosis and mortality using Cox proportional hazards regression models. Results: During a median of 22.83 years of follow-up, 2932 deaths occurred. The risk of all-cause mortality decreased significantly with the healthy lifestyle scores increasing (*p* < 0.001). NAFLD patients with a favorable lifestyle (3 or 4 healthy lifestyle factors) reduced 36% of all-cause mortality and 43% of cardiovascular disease (CVD) mortality compared with those with an unfavorable lifestyle (0 or 1 healthy lifestyle factor) (HR, 0.64 [95% CI, 0.50–0.81], 0.57 [95% CI, 0.37–0.88]). Compared with the non-NAFLD group, the number of NAFLD patients required to adhere to a favorable lifestyle to prevent one cardiovascular disease death in 20 years was fewer (77 vs. 125). Conclusions: For the NAFLD patients, adopting a healthy lifestyle could significantly reduce their risk of death.

## 1. Introduction

Non-alcoholic fatty liver disease (NAFLD) has emerged as the predominant cause of chronic liver disease, with a global prevalence of 25% [1]. NAFLD has become the third most common cause of hepatocellular carcinoma (HCC) in the United States (US), and the number of cases was estimated to increase by 9% on an annual basis [2,3]. In addition, the extrahepatic manifestations of NAFLD lead to a broad disease burden [4]. Thereby an impressive number of prospective cohort studies have reported its association with high risk of all-cause and cause-specific mortality, concentrated in cardiovascular disease (CVD) and cancer-related mortality [5,6,7,8,9]. A study based on the national mortality database of the United States reported that mortality in patients with NAFLD increased from 6.1% per year in 2007–2013 to 11.3% per year in 2013–2016 [10]. To further lower mortality in NAFLD patients, focus should be placed on optimizing behavioral strategies of risk factor management for secondary prevention.

Given the association between NAFLD and metabolic syndrome [11,12], lifestyle modification can improve patients’ life quality and prognosis [1]. Lifestyle-related modifiable factors such as healthy dietary patterns, moderate physical activity, and avoidance of smoking and alcohol are associated with a reduced risk of end-stage liver disease [13,14] and mortality in the general population [15,16,17,18]. Recently, one prospective cohort study has demonstrated that each additional healthy lifestyle factor was associated with 17% lower risk of mortality [19]. However, whether and to what extent a combination of the aforementioned lifestyle factors impacts on the risk of death in NAFLD patients remains unclear, let alone its combined effects on the risk of cause-specific mortality, e.g., cancer mortality and CVD mortality. Additionally, few patients can adhere to lifestyle changes on all sides. So, if there is a single or specific combinations of lifestyle factors which have the lowest mortality, that would refer to a powerful direction for NAFLD patient to alter behaviors.

Here, we used data from the US National Health and Nutrition Examination Survey (NHANES) to assess the joint association of several modifiable lifestyle factors with overall and cause-specific mortality among NAFLD individuals and depict the mortality risk of varied composite modes of lifestyle.

## 2. Methods

### 2.1. Study Participants

This study was based on the NHANES III (1988–1994, the National Center for Health Statistics, the Center for Disease Control and Prevention), which used a complex multistage probability design to recruit a representative sample of civilian, community-dwelling members of the US population. Among 14,797 adult participants (20–74 years) who underwent laboratory testing surveyed by NHANES III, we excluded participants with viral hepatitis (hepatitis B surface antigen positive and/or hepatitis C antibody positive), significant alcohol consumption (men > 30 g/day; women > 20 g/day; one drink contains 14 g of alcohol in the US) [19], iron overload (transferring saturation > 50%) [20], and pregnant women (*n* = 1953). Out of the remaining 12,844 participants, we then excluded those with missing data on poverty income ratio, education level, hepatic ultrasonography, body mass index (BMI), prevalent comorbidities (including hypertension, diabetes, CVD, cancer, emphysema or chronic bronchitis), lifestyle factors (cigarette smoking, alcohol consumption, physical activity, and diet) and deaths (*n* = 3073). Overall, 9771 participants remained in the final analysis.

### 2.2. Definition of NAFLD

The method used for ultrasonography-diagnosed hepatic steatosis has been previously described [21]. Briefly, three board-certified radiologists reviewed the archived gallbladder ultrasound video images to evaluate hepatic steatosis. Referring to the NHANES III procedures manual, hepatic steatosis was assessed using ultrasound images by the following five criteria (https://wwwn.cdc.gov/nchs/data/nhanes3/34a/HGUHS.htm, accessed on 1 January 2011): (i) parenchymal brightness, (ii) liver-to-kidney contrast, (iii) deep beam attenuation, (iv) bright vessel walls, and (v) gallbladder wall definition. Then hepatic steatosis were reported as normal vs. mild, moderate, or severe [22]. In this study, NAFLD was defined as any degree (mild, moderate or severe) of steatosis and absence of heavy alcohol consumption and other causes of chronic liver disease, including viral hepatitis and hemochromatosis.

### 2.3. Assessment of Lifestyle Factors

The structured questionnaire and 24 h dietary review were used to obtain all lifestyle factors. Information on smoking behavior was collected, i.e., smoking status, frequency, amount and years of tobacco smoked. Questions on alcohol consumption included frequency of drinking in the past 12 months, amount of alcohol consumed on a typical drinking day, and past drinking habits. For physical activity, participants were asked about the monthly frequency of walking mile without stop, running or jogging, riding or exercising bicycle, swimming, participating in aerobics or aerobic dancing, participating in other dancing, participating in calisthenics, participating in garden or yard work, lifting weights, and participating in other activities. Intensity ratings were given for each kind of activity, and information on the duration of each activity per time was unavailable. Thus, we added the monthly frequency of all leisure-time physical activities up weighted by their intensity rating. Dietary quality was obtained from 24 h dietary recalls and was assessed by healthy eating index (HEI) scores [23]. The NHANES III HEI score ranges from 0 to 100 and was calculated by adding up 10 equally weighted dietary components (grains, vegetables, fruits, dairy products, meats, total fat, saturated fat, total cholesterol, total sodium, and dietary variety) with a score of 0 to 10. Zero servings were scored as 0 and the maximum score was achieved when the recommended servings were consumed. Higher HEI scores represent a better quality and healthier dietary pattern than lower scores. The NHANES III data used the 1994–1996 version of the HEI [24].

### 2.4. Definition of Low-Risk Lifestyle

Because multiple lifestyle factors are interrelated and associated with mortality, we constructed a healthy lifestyle score that included smoking, drinking, physical activity, and diet based on the recommendations from the World Health Organization and the dietary guidelines in the US and United Kingdom (UK) [25,26,27]. The low-risk group was defined for the following low-risk lifestyle factors: non-smoking (smoked fewer than 100 cigarettes in life, +1 score); non-drinking (had fewer than 12 drinks alcohol in life, +1 score); being physically active (in a sex-specific top third of the physical activity level, +1 score); a healthy diet (HEI scores in the top two-fifths of distribution, +1 score). The healthy lifestyle scores ranged from 0 to 4, then were categorized as favorable (3 or 4 healthy lifestyle factors), intermediate (2 healthy lifestyle factors), and unfavorable (0 or 1 healthy lifestyle factors) lifestyles.

### 2.5. Assessment of Covariates

Covariates were obtained through questionnaires and anthropometric assessment, including age; sex (male or female); self-reported race/ethnicity (non-Hispanic White, non-Hispanic Black, Mexican American, or others); poverty status (poverty income ratio was categorized as ≤0.99 = below the poverty level, or ≥1.00 = at or above the poverty level); education level (less than high school, or high school and above); body mass index (BMI = weight (kg)/(height (m)^2^); history of hypertension (yes or no), diabetes (yes or no) [20], CVD (yes or no), cancer (yes or no), or emphysema or chronic bronchitis (yes or no) [19].

### 2.6. Outcome Ascertainment

All participants over 20 years of age in the NHANES III were followed up for mortality by linkage to the National Death Index (NDI). Follow-up time started from the date of NHANES III participation and extended to 31 December 2015 [22]. For those participants who survived past 31 December 2015, follow-up time was censored. The NHANES III-Linked Mortality File used the Underlying Cause of Death 113 (UCOD_113) code to recode all deaths according to the International Classification of Diseases, 9th Revision (ICD-9) for deaths before 1998 and to ICD-10 for those who died between 1999 and 2015. For the current analysis, all-cause mortality, cardiovascular disease mortality (UCOD_113 55–64, 70) and malignancy mortality (UCOD_113 19–43) were analyzed.

### 2.7. Statistical Analysis

According to the complex sampling design adopted by the NHANES, appropriate sampling weights were used to reconstitute data on a representative population level for the entire United States [28]. Baseline characteristics were compared using the chi-square test for categorical variables and linear regression for continuous variables and described across different lifestyle categories, continuous variables were presented as weighted mean ± standard errors (SE) and categorical variables were presented as numbers with percentages. We used Cox proportional hazard regression models to estimate the hazard ratios (HR) and 95% confidence intervals (95% CI) of all-cause and cause-specific mortality associated with lifestyle categories. Person years were calculated from baseline until the date of death, or end of follow-up, whichever occurred first. The models were adjusted for age at baseline, sex, self-reported race, poverty status, education level, BMI, and history of hypertension, diabetes, CVD, cancer, and emphysema or chronic bronchitis. Data from the NHANES III were analyzed in Poisson regression models to estimate age-, sex-, and race-adjusted rates of all-cause and cause-specific mortality per 100,000 person-years. Absolute risk was calculated as the percentage of all-cause and cause-specific deaths occurring in a given group. We calculated the numbers needed to adhere to a favorable lifestyle to prevent one death by extrapolating the differences of 20-year event rates for given groups. Sensitivity analyses were performed to exclude participants whose deaths occurred during the first one-third year of follow-up. Statistical analyses were performed using R version 4.0.1 (R Foundation for Statistical Computing, Vienna, Austria), and a two-sided *p* value of <0.05 was considered statistically significant.

## 3. Results

Among the 9771 eligible participants, 3578 (34.17%, weighted proportion) were diagnosed with NAFLD (Table 1). The mean age was 42.86 years and 47.01% of the participants were male. For the lifestyle categories, 1528 (16.17%) were categorized as a favorable lifestyle, 2943 (29.01%) followed an intermediate lifestyle, and 5300 (54.81%) with an unfavorable lifestyle. Compared with those with favorable lifestyle, participants with intermediate and unfavorable lifestyle were more likely to be men, fatter, diabetic and had a higher level of education. In addition, these individuals were noted to have a higher plasma concentration of severe hepatic steatosis and alanine aminotransferase (ALT).

During a median follow-up period of 22.83 years, a total of 2932 deaths occurred. The most common cause of death was cardiovascular disease (*n* = 807), followed by cancer (*n* = 702). In Cox proportional hazards analysis, NAFLD patients had a higher all-cause mortality risk than those without NAFLD (HR, 1.17 [95% CI, 1.08–1.26]). However, when more covariates were adjusted, the statistic difference reversed (Table S1). Regarding lifestyle factors, all of the four lifestyle factors were associated with a reduced risk of all-cause mortality (Table S2). When lifestyle factors were combined, each additional healthy lifestyle factor was associated with 20% lower risk of all-cause mortality (HR, 0.80 [95% CI, 0.76–0.85]) (Table S2). The results were identical when the participants were divided into NAFLD (HR, 0.82 [95% CI, 0.76–0.89]) and non-NAFLD group (HR, 0.79 [95% CI, 0.73–0.86]) (Table S3).

**Figure 1:**
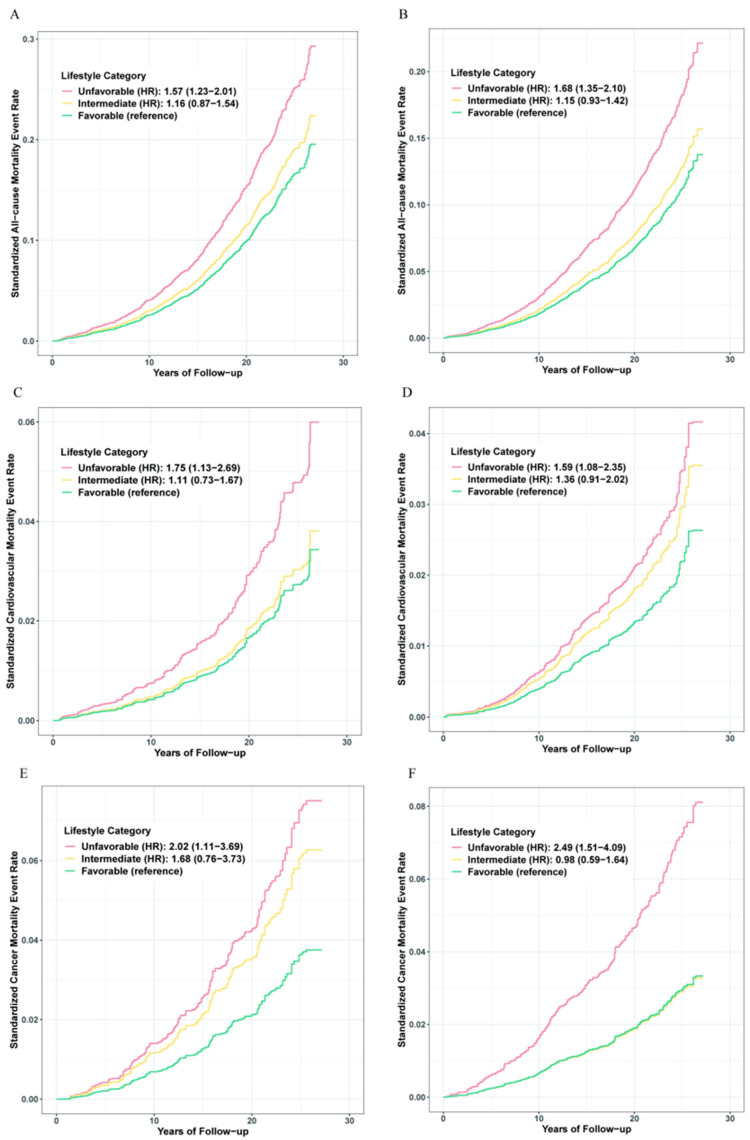
The risk of mortality across lifestyle categories. Standardized rates of mortality events were calculated by using Kaplan-Meier method. HRs and 95% CIs were derived from the Cox regression model. Adjusted for age, sex, ethnicity, poverty status, education level, body mass index, and prevalent comorbidities (including history of hypertension, diabetes, CVD, cancer, chronic bronchitis or emphysema). (**A**,**C**,**E**) for participants with NAFLD; (**B**,**D**,**F**) for participants without NAFLD.

Participants were subsequently divided into three categories: favorable (3 or 4 healthy lifestyle factors), intermediate (2 healthy lifestyle factors), or unfavorable (0 or 1 healthy lifestyle factor). A significant gradient for relative risk of all-cause mortality was observed across lifestyle categories (Figure 1). In the NAFLD group, the adjusted HR for participants in the unfavorable lifestyle category compared with those in the favorable lifestyle category was 1.57 (95% CI, 1.23–2.01) for all-cause mortality, 1.75 (95% CI, 1.13–2.69) for cardiovascular disease mortality, and 2.02 (95% CI, 1.11–3.69) for cancer disease mortality. An equal effect was also present in the non-NAFLD group.

Standardized rates of mortality events were calculated by using Kaplan–Meier method. HRs and 95% CIs were derived from the Cox regression model. Adjusted for age, sex, ethnicity, poverty status, education level, body mass index, and prevalent comorbidities (including history of hypertension, diabetes, CVD, cancer, chronic bronchitis or emphysema), with A, C, E for participants with NAFLD and B, D, F for participants without NAFLD.

Further analysis exhibited the benefit of a favorable lifestyle for risk reduction of all-cause and cause-specific mortality (Table 2). The number of participants with NAFLD needed to adhering to a favorable lifestyle to prevent one death in 20 years were 16 for all-cause mortality, 77 for cardiovascular disease mortality, and 45 for cancer disease mortality. Noteworthy, because of differences in baseline CVD risk, the effect of adhering to a healthy lifestyle on risk reduction of cardiovascular mortality was greater in the NAFLD group compared with the non-NAFLD group (HR, 0.63 [95% CI, 0.40–1.01] vs. HR, 0.85 [95% CI, 0.57–1.28] for adhering to an intermediate lifestyle; HR, 0.57 [95% CI, 0.37–0.88] vs. HR, 0.63 [95% CI, 0.43–0.93] for adhering to a healthy lifestyle), and fewer NAFLD participants were needed to adhere to a favorable lifestyle to prevent one cardiovascular disease death in 20 years (77 vs. 125).

**Figure 2:**
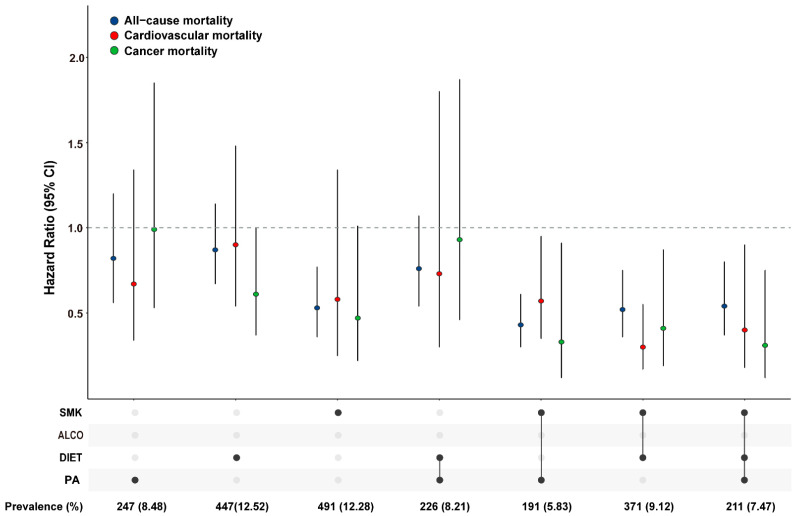
Association between combinations of the healthy lifestyle factors and risk of mortality in NAFLD patients.

To identify specific combinations of lifestyle factors with the lowest mortality, we conducted analyses according to varied composite modes of healthy lifestyle factors compared to zero factor. Only those combinations with exposure rate ≥ 5% were taken into consideration. Multivariable analyses revealed a gradient distribution of the protection effect of healthy lifestyle on the risk of all-cause and cause-specific mortality (Figure 2). Of which, we found the protective effect of a single non-smoking lifestyle was equivalent to that of two or three healthy factor combinations in both NAFLD and non-NAFLD groups (Figure S1; Table S7).

HRs and 95% CIs were derived from the Cox regression model adjusting for age, sex, ethnicity, poverty status, education level, body mass index, and prevalent comorbidities (including history of hypertension, diabetes, CVD, cancer, chronic bronchitis or emphysema). Combinations of the healthy lifestyle factors (proportion > 5%).

## 4. Discussion

The main finding in this large, nationally representative, population-based study was a protective effect among NAFLD individuals adhering to a healthy lifestyle, e.g., abstaining from smoking and drinking, being physically active, eating a healthy diet, on the risk reduction of all-cause mortality, largely acting on the cardiovascular complications. Notably, among the most common lifestyle factor combinations, the effect of risk reduction on mortality was particularly strong when smoking was avoided.

Previous studies have estimated the clustering effect of lifestyle related risk factors on risk reduction of deaths with and without chronic diseases. A pooled meta-analysis of 15 studies estimated that nearly 60% of premature deaths could be attributed to lifestyle behaviors in the general population [29]. Yanping et al. [30] found that adherence to a healthy lifestyle at mid-life is associated with a longer life expectancy free of major chronic diseases. A prospective analysis from the UK Biobank database revealed that engaging in a healthier lifestyle was associated with longer life for individuals with multimorbidity [29]. Similarly, there has been a series of epidemiology studies evaluated the association between healthy lifestyle with the risk of deaths for other chronic diseases, i.e., diabetes [30], cancer [14], inflammatory disease [31], and CVD [32]. Our study extends the previous knowledge of the association even for fatty liver disease. In this study, the results showed that the risk of mortality was 36% lower in the healthiest group than in the unhealthiest group in people with NAFLD and a risk 40% lower in those without NAFLD. Additionally, when a cumulative score was applied, we observed that the risk of death declined in a gradient. Hence, the extent to which modifiable lifestyle behaviors impact mortality in the NAFLD individuals is the principal finding in the present study.

When comparing associations between lifestyle factors and cause-specific mortality, we observed some differences. Although the healthiest group indicated lower risk of cancer and CVD mortality in those with and without NAFLD, it appeared to have a stronger impact on CVD mortality for the NAFLD individuals. This difference in effect has been postulated to be due to the differential effect intensity of lifestyle on CVD and cancer, which was in accordance with previous epidemiology studies that the most common causes of death are CVD, followed by extrahepatic malignancies and liver-related complications [33]. Therefore, modifiable lifestyle behaviors specifically impact CVD related mortality for NAFLD individuals is the secondary finding in this study.

Regarding the individual lifestyle factor chosen in this study, we found that not smoking had the largest impact on risk reduction of mortality for those with and without NAFLD. This finding is in agreement with studies from the general population [34] and those with other chronic disease [35]. Two previous studies also estimated the effect of smoking on life expectancies separately from other lifestyle factors [36,37]. Smoking has a somewhat larger impact on life expectancy than physical activity or obesity. Anyway, as few patients can adhere to lifestyle changes on all sides, our results point out a cost-effective strategy for NAFLD patients, that is, smoking avoidance.

Taking advantages of NHANES, a large, nationwide representative survey, our study has the unique feature of assessing the associations, and the results can be generalized more widely to the US population. However, our study also had several limitations. First, we used recalled data on lifestyle behaviors, so that measurement errors are inevitable. Second, even when we controlled for a wide range of potential confounders, there may be unmeasured confounders. Third, the present populations are limited to the US population, and the results may not be generalizable to other ethnic and racial people.

## 5. Conclusions

In conclusion, adherence to key lifestyle factors was associated with lower mortality in NAFLD patients, largely impacting CVD-related mortality. The results of this study can be a useful tool to help the general public and patients with NAFLD to understand the importance of maintaining a healthy lifestyle. Even though adoption of any of these behaviors would likely improve survival rate, smoking status is the major contributor.

## Figures and Tables

**Table 1:** Baseline characteristics of the study population according to healthy lifestyle categories.

Characteristics	Total Population	Unfavorable Lifestyle	Intermediate Lifestyle	Favorable Lifestyle	*p* Value
	*n* = 9771	*n* = 5300	*n* = 2943	*n* = 1528	
**Mean age (years)**	42.86 ± 0.42	42.14 ± 0.44	43.87 ± 0.52	43.50 ± 0.83	0.012
**Gender (%)**					<0.001
Male	4366 (47.01)	2854 (54.43)	1116 (42.12)	396 (30.65)	
Female	5405 (52.99)	2446 (45.57)	1827 (57.88)	1132 (69.35)	
**Ethnicity (%)**					<0.001
Non-Hispanic White	3875 (77.55)	2150 (79.93)	1096 (74.87)	629 (74.32)	
Non-Hispanic Black	2766 (9.97)	1585 (10.19)	809 (10.63)	372 (8.01)	
Mexican American	2728 (4.87)	1407 (4.44)	897 (5.69)	424 (4.84)	
Others	402 (7.61)	158 (5.44)	141 (8.81)	103 (12.83)	
**Poverty status (%)**					0.007
Yes	2185 (11.47)	1239 (12.65)	614 (9.70)	332 (10.66)	
No	7586 (88.53)	4061 (87.35)	2329 (90.30)	1196 (89.34)	
**Education**					<0.001
Less than high school	3517 (21.59)	2094 (25.12)	934 (17.91)	489 (16.22)	
High school or above	6254 (78.41)	3206 (74.88)	2009 (82.09)	1039 (83.78)	
**Body mass index (kg/m^2^)**	26.70 ± 0.14	27.07 ± 0.16	26.55 ± 0.21	25.76 ± 0.19	<0.001
Waist circumference (cm) *	91.90 ± 0.30	93.64 ± 0.35	90.81 ± 0.52	87.93 ± 0.49	<0.001
Total cholesterol (mg/dL) *	203.60 ± 0.83	203.80 ± 0.96	203.49 ± 1.44	203.14 ± 1.72	0.925
HDL-cholesterol (mg/dL) *	49.74 ± 0.38	47.89 ± 0.41	51.67 ± 0.55	52.60 ± 0.56	<0.001
Fasting glucose (mg/dL) *	98.21 ± 0.55	98.63 ± 0.57	98.83 ± 0.92	95.70 ± 0.86	0.003
Hemoglobin A1c (%) *	5.36 ± 0.03	5.40 ± 0.02	5.33 ± 0.04	5.29 ± 0.04	0.005
**Alanine aminotransferase** (IU/L) *	17.52 ± 0.39	17.68 ± 0.40	17.76 ± 0.57	16.56 ± 0.53	0.027
**Aspartate aminotransferase** (IU/L) *	20.82 ± 0.14	20.46 ± 0.17	21.55 ± 0.28	20.71 ± 0.27	0.002
**Self-reported comorbidities (%)**					
Hypertension	3154 (27.48)	1748 (28.11)	934 (27.63)	472 (25.06)	0.298
Diabetes	1018 (7.06)	576 (7.49)	294 (6.94)	148 (5.81)	0.196
CVD	600 (4.42)	376 (4.63)	168 (5.14)	56 (2.43)	0.017
Cancer	537 (6.69)	272 (6.19)	179 (8.20)	86 (5.68)	0.049
Emphysema or chronic bronchitis	645 (7.24)	430 (9.10)	155 (5.83)	60 (3.44)	<0.001
**NAFLD (%)**	3578 (34.17)	1992 (35.27)	1049 (33.71)	537 (31.30)	0.041
**Hepatic Steatosis**					0.069
Normal	6193 (65.83)	3308 (64.73)	1894 (66.29)	991 (68.70)	
Mild	1344 (13.91)	706 (13.75)	409 (13.37)	229 (15.44)	
Moderate	1498 (13.54)	855 (14.29)	435 (13.81)	208 (10.49)	
Severe	736 (6.72)	431 (7.22)	205 (6.53)	100 (5.37)	

All estimates accounted for complex survey designs. For categorical variables, *p* value was calculated by Rao-Scott χ^2^ test, which is design adjusted version of Pearson χ^2^ test. For continuous variables, liner regression adjusted for sampling weights was used to calculate *p* value. * Missing data: Waist circumference (*n* = 220); Total cholesterol (*n* = 146); HDL-cholesterol (*n* = 215); Fasting glucose (*n* = 23); Hemoglobin A1c (*n* = 58); Alanine aminotransferase (*n* = 240); Aspartate aminotransferase (*n* = 240).

**Table 2:** Risk of mortality according to lifestyle categories.

Healthy Lifestyle Category	NAFLD			No NAFLD		
	Unfavorable Lifestyle	Intermediate Lifestyle	Favorable Lifestyle	Unfavorable Lifestyle	Intermediate Lifestyle	Favorable Lifestyle
**All-cause mortality**						
No. of total	1992 (56.57%)	1049 (28.62%)	537 (14.81%)	3308 (53.90%)	1894 (29.22%)	991 (16.88%)
No. of cases/ Person-years	781/39498	353/21491	149/11289	962/69281	444/41278	243/21326
HR (95% CI) ^a^	1 [Reference]	0.73 (0.59–0.91)	0.64 (0.50–0.81)	1 [Reference]	0.68 (0.57–0.82)	0.60 (0.48–0.74)
*p* value ^a^		0.006	<0.001		<0.001	<0.001
*p* value for trend ^a^	<0.001			<0.001		
Absolute risk, % (95% CI)	39.21 (37.06–41.39)	33.65 (30.81–36.61)	27.75 (24.04–31.78)	29.08 (27.54–30.67)	23.44 (21.56–25.43)	24.52 (21.90–27.35)
Incidence rate per 1000 PYs (95% CI) ^b^	13.37 (12.23–14.61)	10.60 (9.39–11.96)	8.51 (7.15–10.13)	8.67 (7.98–9.41)	6.16 (5.51–6.87)	5.81 (5.03–6.70)
Numbers needed -20 years ^c^		22	16		27	21
**Cardiovascular mortality**						
No. of total	1992 (56.57%)	1049 (28.62%)	537 (14.81%)	3308 (53.90%)	1894 (29.22%)	991 (16.88%)
No. of cases/ Person years	225/39498	95/21491	44/11289	248/69281	132/41278	63/21326
HR (95% CI) ^a^	1 [Reference]	0.63 (0.40–1.01)	0.57 (0.37–0.88)	1 [Reference]	0.85 (0.57–1.28)	0.63 (0.43–0.93)
*p* value ^a^		0.055	0.011		0.438	0.020
*p* value for trend ^a^	0.018			0.045		
Absolute risk, % (95% CI)	11.30 (9.96–12.79)	9.06 (7.42–11.00)	8.19 (6.08–10.93)	7.50 (6.63–8.46)	6.97 (5.88–8.23)	6.36 (4.96–8.11)
Incidence rate per 1000 PYs (95% CI) ^b^	3.05 (2.52–3.68)	2.25 (1.76–2.89)	2.02 (1.45–2.83)	1.66 (1.37–1.99)	1.30 (1.04–1.64)	1.04 (0.77–1.41)
Numbers needed -20 years ^c^		90	77		311	125
**Cancer mortality**						
No. of total	1992 (56.57%)	1049 (28.62%)	537 (14.81%)	3308 (53.90%)	1894 (29.22%)	991 (16.88%)
No. of cases/ Person years	178/39498	80/21491	31/11289	269/69281	89/41278	55/21326
HR (95% CI) ^a^	1 [Reference]	0.83 (0.54–1.27)	0.49 (0.27–0.90)	1 [Reference]	0.39 (0.28–0.55)	0.40 (0.24–0.66)
*p* value ^a^		0.394	0.022		<0.001	<0.001
*p* value for trend ^a^	0.007			<0.001		
Absolute risk, % (95% CI)	8.94 (7.74–10.30)	7.63 (6.13–9.44)	5.77 (4.02–8.18)	8.13 (7.23–9.13)	4.70 (3.81–5.78)	5.55 (4.24–7.21)
Incidence rate per 1000 PYs (95% CI) ^b^	3.38 (2.84–4.04)	2.68 (2.10–3.43)	1.97 (1.35–2.87)	2.70 (2.32–3.13)	1.36 (1.08–1.72)	1.43 (1.07–1.92)
Numbers needed -20 years ^c^		135	45		35	35

^a^ Adjusted for age, sex, ethnicity, poverty status, education level, body mass index, and prevalent comorbidities (including history of hypertension, diabetes, CVD, cancer, chronic bronchitis or emphysema). ^b^ Incidence rate for liver cancer are adjusted for age at baseline, gender, and ethnicity. ^c^ The numbers needed to adhere to a healthy lifestyle to prevent one death in 20 years. To minimize potential bias due to subclinical status, sensitivity analyses were performed to exclude participants with outcomes (all-cause mortality/cardiovascular mortality/cancer mortality) occurred during the 1st year/3rd years of follow-up. These sensitivity analyses did not substantially alter the risk estimates (Tables S4 and S5). In the NAFLD group, when grouped according to baseline characteristics, we also observed the protective effect of healthy lifestyle (Table S6).

## Data Availability

The datasets used and analyzed during the current study are available at https://wwwn.cdc.gov/nchs/nhanes/nhanes3/DataFiles.aspx (accessed on 1 March 2011).

## Supplementary Materials

The following supporting information can be downloaded at: https://www.mdpi.com/article/10.3390/nu14183785/s1. Figure S1. Association between combinations of the healthy lifestyle factors (proportion > 5%) and risk of all-cause mortality/cardiovascular mortality/cancer mortality in the subcohort without NAFLD; Table S1. Association between healthy lifestyle categories or NAFLD status and all-cause mortality; Table S2. Age, sex, ethnicity-adjusted and multivariate hazard ratio of risk factor for the all-cause mortality according to healthy lifestyle factors and individual components; Table S3. Association between the healthy lifestyle factors or individual components and all-cause mortality according to NAFLD status; Table S4. Risk of mortality according to lifestyle categories after excluding incidents during the first year of follow-up; Table S5. Risk of mortality according to lifestyle categories after excluding incidents during the first 3 years of follow-up; Table S6. Multivariable-adjusted HRs (95% CIs) for mortality by healthy lifestyle categories according to baseline characteristics; Table S7. Association between combinations of the healthy lifestyle factors (prevalence > 5%) and risk of liver cancer.
