# Supplementary material for: Healthy Lifestyle Is Associated with Reduced Mortality in Patients with Non-Alcoholic Fatty Liver Disease
Source: Nutrients. 2022 Sep 14;14(18):3785. doi: 10.3390/nu14183785 (PMC9506586; doi:10.3390/nu14183785)

Healthy Lifestyle Is Associated with Reduced Mortality in Patients with Non-Alcoholic Fatty Liver Disease

Chengxiao Yu [1,2,†], Jiaxin Gao [1,†], Xinyuan Ge [1], Xiao Wang [3], Yuqing Ding [1], Ting Tian [1], Xin Xu [1], Wen Guo [4], Quanrongzi Wang [5], Zijun Ge [6], Tao Jiang [1], Qun Zhang [4,*] and Ci Song [1,7,*]

Contents

- Table S1. Association between healthy lifestyle categories or NAFLD status and all-cause mortality.
- Table S2. Age, sex, ethnicity-adjusted and multivariate hazard ratio of risk factor for the all-cause mortality according to healthy lifestyle factors and individual components.
- Table S3. Association between the healthy lifestyle factors or individual components and all-cause mortality according to NAFLD status.
- Table S4. Risk of mortality according to lifestyle categories after excluding incidents during the first year of follow-up.
- Table S5. Risk of mortality according to lifestyle categories after excluding incidents during the first three years of follow-up.
- Table S6. Multivariable-adjusted HRs (95% CIs) for mortality by healthy lifestyle categories according to baseline characteristics.
- Table S7. Association between combinations of the healthy lifestyle factors (Prevalence >5%) and risk of liver cancer.
- Figure S1. Association between combinations of the healthy lifestyle factors (Proportion >5%) and risk of all-cause mortality/cardiovascular mortality/cancer mortality in the sub cohort without NAFLD.

Table S1. Association between healthy lifestyle categories or NAFLD status and all-cause mortality.

	Age, sex, ethnicity-adjusted		Multivariate model	
	**HR(95% CI) [a]**	***P* value**	**HR(95% CI) [b]**	***P* value**
**Healthy Lifestyle Category**				
Unfavorable Lifestyle	1 [Reference]		1 [Reference]	
Intermediate Lifestyle	0.67 (0.58-0.76)	<0.001	0.70 (0.62-0.80)	<0.001
Favorable Lifestyle	0.54 (0.45-0.63)	<0.001	0.60 (0.51-0.71)	<0.001
**NAFLD**				
No NAFLD	1 [Reference]		1 [Reference]	
NAFLD	1.17 (1.08-1.26)	<0.001	1.07 (0.98-1.16)	0.115
**Hepatic Steatosis**				
Normal	1 [Reference]		1 [Reference]	
Mild	1.16 (1.01-1.33)	0.042	1.09 (0.95-1.25)	0.196
Moderate	1.17 (1.06-1.29)	0.001	1.03 (0.93-1.13)	0.581
Severe	1.18 (0.98-1.42)	0.074	1.12 (0.94-1.33)	0.217

[a] Analyses were adjusted for age, sex, ethnicity.

[b] Analyses were adjusted for age, sex, ethnicity, poverty status, education level, body mass index, and prevalent comorbidities (including history of CVD, cancer, chronic bronchitis or emphysema).

Table S2. Age, sex, ethnicity-adjusted and multivariate hazard ratio of risk factor for the all-cause mortality according to healthy lifestyle factors and individual components.

	Age, sex, ethnicity-adjusted		Multivariate model	
	HR(95% CI) [a]	*P* value	HR(95% CI) [b]	*P* value
**Healthy Lifestyle Score**				
Score 0	1 [Reference]		1 [Reference]	
Score 1	0.74 (0.65-0.84)	<0.001	0.78 (0.68-0.89)	<0.001
Score 2	0.55 (0.47-0.65)	<0.001	0.60 (0.51-0.71)	<0.001
Score 3	0.45 (0.38-0.54)	<0.001	0.53 (0.44-0.64)	<0.001
Score 4	0.40 (0.30-0.53)	<0.001	0.49 (0.37-0.66)	<0.001
One-point increase in the healthy lifestyle score	0.77 (0.73-0.81)	<0.001	0.80 (0.76-0.85)	<0.001
**Individual component of healthy lifestyle categories**				
**Ever smoked 100 cigarettes**				
Yes	1 [Reference]		1 [Reference]	
No	0.57 (0.51-0.63)	<0.001	0.60 (0.54-0.66)	<0.001
**Ever had 12 drinks**				
Yes	1 [Reference]		1 [Reference]	
No	0.92 (0.77-1.11)	0.939	0.85 (0.72-1.01)	0.060
**Regular physical activity**				
No	1 [Reference]		1 [Reference]	
Yes	0.81 (0.72-0.90)	<0.001	0.90 (0.81-0.99)	0.039
**Healthy dietary pattern**				
No	1 [Reference]		1 [Reference]	
Yes	0.74 (0.67-0.83)	<0.001	0.82 (0.72-0.92)	0.001

[a] Analyses were adjusted for age, sex, ethnicity.

[b] Analyses were adjusted for age, sex, ethnicity, poverty status, education level, body mass index, and prevalent comorbidities (including history of hypertension, diabetes, CVD, cancer, chronic bronchitis, or emphysema).

Table S3. Association between the healthy lifestyle factors or individual components and all-cause mortality according to NAFLD status.

	NAFLD		No NAFLD	
	**HR(95% CI) [a]**	***P* value**	**HR(95% CI) [a]**	***P* value**
**Healthy Lifestyle Category**				
Score 0	1 [Reference]		1 [Reference]	
Score 1	0.78 (0.62-0.98)	0.036	0.79 (0.66-0.94)	0.009
Score 2	0.63 (0.48-0.82)	0.001	0.59 (0.47-0.74)	<0.001
Score 3	0.52 (0.39-0.70)	<0.001	0.53 (0.41-0.69)	<0.001
Score 4	0.62 (0.38-1.01)	0.057	0.39 (0.25-0.61)	<0.001
One-point increase in the healthy lifestyle score	0.82 (0.76-0.89)	<0.001	0.79 (0.73-0.86)	<0.001
**Individual component of healthy lifestyle categories**				
**Ever smoked 100 cigarettes**				
Yes	1 [Reference]		1 [Reference]	
No	0.59 (0.50-0.70)	<0.001	0.60 (0.52-0.69)	<0.001
**Ever had 12 drinks**				
Yes	1 [Reference]		1 [Reference]	
No	0.88 (0.69-1.13)	0.309	0.79 (0.66-0.96)	0.016
**Regular physical activity**				
No	1 [Reference]		1 [Reference]	
Yes	0.89 (0.72-1.10)	0.281	0.93 (0.83-1.05)	0.250
**Healthy dietary pattern**				
No	1 [Reference]		1 [Reference]	
Yes	0.91 (0.77-1.09)	0.300	0.75 (0.65-0.88)	<0.001

[a] Analyses were adjusted for age, sex, ethnicity, poverty status, education level, body mass index, and prevalent comorbidities (including history of hypertension, diabetes, CVD, cancer,chronic bronchitis, or emphysema).

Table S4. Risk of mortality according to lifestyle categories after excluding incidents during the first year of follow-up.

Healthy Lifestyle Category	NAFLD			No NAFLD		
	Unfavorable Lifestyle	Intermediate Lifestyle	Favorable Lifestyle	Unfavorable Lifestyle	Intermediate Lifestyle	Favorable Lifestyle
**All-cause mortality**						
No. of total	1980 (56.47%)	1046 (28.73%)	536 (14.80%)	3283 (53.83%)	1889 (29.26%)	987 (16.92%)
No. of cases/ Person years	769/39490	350/21491	148/11289	937/69267	439/41275	239/21325
HR (95% CI) [a]	1 [Reference]	0.74 (0.60-0.93)	0.62 (0.49-0.79)	1 [Reference]	0.69 (0.57-0.82)	0.60 (0.48-0.75)
P value [a]		0.009	<0.001		<0.001	<0.001
P value for trend [a]		<0.001			<0.001	
Absolute risk, % (95% CI)	38.84 (36.69-41.03)	33.46 (30.62-36.42)	27.61 (23.91-31.64)	28.54 (27.01-30.13)	23.24 (21.36-25.22)	24.21 (21.60-27.04)
Incidence rate per 1000 PYs (95% CI) [b]	13.12 (11.99-14.35)	10.45 (9.25-11.79)	8.39 (7.04-10.00)	8.44 (7.77-9.17)	6.08 (5.44-6.79)	5.70 (4.94-6.59)
Numbers needed -20 years [c]		24	16		28	21
**Cardiovascular mortality**						
No. of total	1985 (56.51%)	1049 (28.71%)	536 (14.78%)	3296 (53.87%)	1892 (29.24%)	990 (16.88%)
No. of cases/ Person years	218/39493	95/21491	43/11289	236/69275	130/41277	62/21326
HR (95% CI) [a]	1 [Reference]	0.66 (0.41-1.06)	0.52 (0.33-0.82)	1 [Reference]	0.86 (0.56-1.31)	0.61 (0.41-0.91)
P value [a]		0.088	0.005		0.474	0.014
P value for trend [a]		0.014			0.041	
Absolute risk, % (95% CI)	10.98 (9.66-12.46)	9.06 (7.42-11.00)	8.02 (5.93-10.74)	7.16 (6.31-8.11)	6.87 (5.79-8.13)	6.26 (4.87-8.00)
Incidence rate per 1000 PYs (95% CI) [b]	2.95 (2.43-3.57)	2.24 (1.74-2.87)	1.95 (1.39-2.74)	1.61 (1.33-1.94)	1.31 (1.04-1.64)	1.04 (0.77-1.41)
Numbers needed -20 years [c]		105	74		333	122
**Cancer mortality**						

No. of total	1990 (56.56%)	1049 (28.62%)	537 (14.81%)	3303 (53.86%)	1894 (29.24%)	990 (16.89%)
No. of cases/ Person years	176/39496	80/21491	31/11289	264/69278	89/41278	54/21326
HR (95% CI) [a]	1 [Reference]	0.83 (0.55-1.27)	0.49 (0.27-0.90)	1 [Reference]	0.40 (0.29-0.57)	0.41 (0.25-0.68)
P value [a]		0.396	0.022		<0.001	<0.001
P value for trend [a]		0.007			<0.001	
Absolute risk, % (95% CI)	8.84 (7.65-10.20)	7.63 (6.13-9.44)	5.77 (4.02-8.18)	7.99 (7.10-8.98)	4.70 (3.81-5.78)	5.45 (4.16-7.11)
Incidence rate per 1000 PYs (95% CI) [b]	3.33 (2.79-3.97)	2.67 (2.09-3.41)	1.96 (1.35-2.85)	2.66 (2.29-3.09)	1.37 (1.08-1.73)	1.42 (1.06-1.91)
Numbers needed -20 years [c]		136	45		35	36

[a] Adjusted for age, sex, ethnicity, poverty status, education level, body mass index, and prevalent comorbidities (including history of hypertension, diabetes, CVD, cancer, chronic bronchitis, or emphysema).

[b] Incidence rate for liver cancer are adjusted for age at baseline, gender, and ethnicity.

[c] The numbers needed to adhere to a healthy lifestyle to prevent one death in 20 years.

Table S5. Risk of mortality according to lifestyle categories after excluding incidents during the first three years of follow-up.

Healthy Lifestyle Category	NAFLD			No NAFLD		
	Unfavorable Lifestyle	Intermediate Lifestyle	Favorable Lifestyle	Unfavorable Lifestyle	Intermediate Lifestyle	Favorable Lifestyle
**All-cause mortality**						
No. of total	1942 (56.51%)	1025 (28.56%)	532 (14.92%)	3234 (53.73%)	1875 (29.32%)	980 (16.95%)
No. of cases/ Person years	731/39418	329/21448	144/11281	888/69165	425/41246	232/21309
HR (95% CI) [a]	1 [Reference]	0.72 (0.57-0.91)	0.62 (0.49-0.78)	1 [Reference]	0.69 (0.58-0.83)	0.59 (0.48-0.73)
P value [a]		0.007	<0.001		<0.001	<0.001
P value for trend [a]		<0.001			<0.001	
Absolute risk, % (95% CI)	37.64 (35.49-39.84)	32.10 (29.26-35.07)	27.07 (23.38-31.10)	27.46 (25.93-29.04)	22.67 (20.80-24.64)	23.67 (21.07-26.49)
Incidence rate per 1000 PYs (95% CI) [b]	12.40 (11.30-13.60)	9.73 (8.59-11.03)	8.03 (6.72-9.60)	8.00 (7.35-8.71)	5.86 (5.23-6.56)	5.50 (4.75-6.37)
Numbers needed -20 years [c]		23	17		30	22
**Cardiovascular mortality**						
No. of total	1968 (56.45%)	1043 (28.75%)	535 (14.80%)	3279 (53.85%)	1890 (29.26%)	988 (16.89%)
No. of cases/ Person years	201/39460	89/21479	42/11286	219/69239	128/41273	60/21321
HR (95% CI) [a]	1 [Reference]	0.67 (0.40-1.10)	0.51 (0.32-0.81)	1 [Reference]	0.88 (0.57-1.35)	0.61 (0.41-0.92)
P value [a]		0.115	0.005		0.547	0.018
P value for trend [a]		0.015			0.051	
Absolute risk, % (95% CI)	10.21 (8.93-11.66)	8.53 (6.94-10.44)	7.85 (5.78-10.55)	6.68 (5.86-7.60)	6.77 (5.70-8.02)	6.07 (4.70-7.80)
Incidence rate per 1000 PYs (95% CI) [b]	2.78 (2.28-3.38)	2.15 (1.67-2.78)	1.96 (1.39-2.76)	1.51 (1.25-1.84)	1.30 (1.03-1.64)	1.01 (0.74-1.38)
Numbers needed -20 years [c]		115	78		401	128
**Cancer mortality**						

No. of total	1985 (56.66%)	1044 (28.51%)	534 (14.83%)	3293 (53.85%)	1889 (29.22%)	987 (16.93%)
No. of cases/ Person years	171/39484	75/21482	28/11284	254/69261	84/41266	51/21320
HR (95% CI) [a]	1 [Reference]	0.76 (0.49-1.18)	0.47 (0.25-0.87)	1 [Reference]	0.39 (0.28-0.55)	0.42 (0.25-0.70)
P value [a]		0.221	0.017		<0.001	0.001
P value for trend [a]		0.003			<0.001	
Absolute risk, % (95% CI)	8.61 (7.44-9.96)	7.18 (5.73-8.96)	5.24 (3.58-7.58)	7.71 (6.384-8.69)	4.45 (3.58-5.50)	5.17 (3.91-6.79)
Incidence rate per 1000 PYs (95% CI) [b]	3.26 (2.72-3.90)	2.51 (1.95-3.23)	1.76 (1.19-2.61)	2.60 (2.23-3.03)	1.32 (1.04-1.67)	1.37 (1.01-1.85)
Numbers needed -20 years [c]		98	44		35	37

[a] Adjusted for age, sex, ethnicity, poverty status, education level, body mass index, and prevalent comorbidities (including history of hypertension, diabetes, CVD, cancer,chronic bronchitis, or emphysema).

[b] Incidence rate for liver cancer are adjusted for age at baseline, gender, and ethnicity.

[c] The numbers needed to adhere to a healthy lifestyle to prevent one death in 20 years.

Table S6. Multivariable-adjusted HRs (95% CIs) for mortality by healthy lifestyle categories according to baseline characteristics.

Subgroups		No. of total	No. of All-cause mortality	No. of Cardiovascular mortality	No. of Cancer mortality	Person years	All-cause mortality		Cardiovascular mortality		Cancer mortality	
							HR(95%CI) [a]	P value [a]	HR(95%CI) [a]	P value [a]	HR(95%CI) [a]	P value [a]
**Age at baseline, years**												
<50	Unfavorable	1,120	175	40	46	25,267	1 [Reference]		1 [Reference]		1 [Reference]	
	Intermediate	583	69	16	19	13,249	0.96 (0.59-1.56)	0.862	1.72 (0.55-5.36)	0.346	0.81 (0.35-1.86)	0.621
	Favorable	297	20	7	3	6,787	0.42 (0.18-1.01)	0.051	0.40 (0.14-1.14)	0.085	0.46 (0.11-2.01)	0.302
≥50	Unfavorable	872	606	185	132	14,231	1 [Reference]		1 [Reference]		1 [Reference]	
	Intermediate	466	284	79	61	8,242	0.84 (0.69-1.02)	0.078	0.69 (0.48-1.01)	0.055	1.00 (0.65-1.54)	0.994
	Favorable	240	129	37	28	4,503	0.84 (0.64-1.11)	0.222	0.81 (0.49-1.32)	0.392	0.64 (0.36-1.15)	0.136
**Gender**												
Male	Unfavorable	1,119	505	160	108	21,399	1 [Reference]		1 [Reference]		1 [Reference]	
	Intermediate	411	143	36	33	8,389	0.69 (0.52-0.92)	0.011	0.41 (0.26-0.64)	<0.001	0.86 (0.47-1.58)	0.622
	Favorable	122	33	9	10	2,513	0.66	0.094	0.72	0.433	0.71	0.384

							(0.40-1.07)		(0.32-1.63)		(0.32-1.54)	
	Unfavorable	873	276	65	70	18,098	1 [Reference]		1 [Reference]		1 [Reference]	
Female	Intermediate	638	210	59	47	13,103	0.80 (0.63-1.01)	0.062	1.01 (0.59-1.75)	0.963	0.76 (0.47-1.21)	0.247
	Favorable	415	116	35	21	8,777	0.66 (0.52-0.85)	0.001	0.65 (0.39-1.08)	0.096	0.44 (0.22-0.88)	0.021
**Ethnicity**												
	Unfavorable	779	341	97	82	15,178	1 [Reference]		1 [Reference]		1 [Reference]	
Non-Hispanic white	Intermediate	384	169	50	41	7,521	0.74 (0.57-0.96)	0.024	0.65 (0.38-1.10)	0.107	0.85 (0.52-1.39)	0.526
	Favorable	206	77	20	16	4,209	0.57 (0.43-0.75)	<0.001	0.51 (0.31-0.83)	0.007	0.43 (0.22-0.83)	0.013
	Unfavorable	487	208	54	45	9,375	1 [Reference]		1 [Reference]		1 [Reference]	
Non-Hispanic black	Intermediate	246	62	13	12	5,303	0.52 (0.37-0.74)	<0.001	0.56 (0.28-1.12)	0.102	0.49 (0.24-0.99)	0.047
	Favorable	124	28	12	4	2,593	0.72 (0.45-1.15)	0.167	1.36 (0.60-3.06)	0.458	0.43 (0.16-1.15)	0.092
	Unfavorable	660	217	69	49	13,569	1 [Reference]		1 [Reference]		1 [Reference]	
Mexican-American	Intermediate	376	117	32	26	7,690	1.20 (0.91-1.58)	0.204	0.83 (0.43-1.60)	0.571	0.94 (0.49-1.80)	0.860
	Favorable	174	37	10	10	3,780	0.65 (0.40-1.05)	0.079	0.62 (0.21-1.81)	0.382	0.45 (0.18-1.15)	0.096

	Unfavorable	66	15	5	2	1,377	1 [Reference]		1 [Reference]		1 [Reference]	
Others	Intermediate	43	5	0	1	978	0.56 (0.20-1.52)	0.253	(-)	(-)	(-)	(-)
	Favorable	33	7	2	1	708	1.64 (0.39-6.95)	0.503	(-)	(-)	(-)	(-)
**Poverty status**												
	Unfavorable	499	201	53	49	9,670	1 [Reference]		1 [Reference]		1 [Reference]	
Yes	Intermediate	227	73	18	15	4,692	0.39 (0.26-0.57)	<0.001	0.56 (0.25-1.29)	0.173	0.28 (0.10-0.77)	0.014
	Favorable	147	36	11	7	3,075	0.51 (0.32-0.81)	0.005	0.57 (0.20-1.64)	0.300	0.18 (0.04-0.75)	0.018
	Unfavorable	1,493	580	172	129	29,828	1 [Reference]		1 [Reference]		1 [Reference]	
No	Intermediate	822	280	77	65	16,799	0.81 (0.63-1.03)	0.086	0.68 (0.42-1.08)	0.101	1.01 (0.65-1.58)	0.965
	Favorable	390	113	33	24	8,214	0.65 (0.51-0.84)	0.001	0.60 (0.37-0.96)	0.034	0.58 (0.32-1.07)	0.080
**Education**												
	Unfavorable	909	457	143	98	16,916	1 [Reference]		1 [Reference]		1 [Reference]	
Less than high school	Intermediate	390	178	47	39	7,480	0.73 (0.52-1.02)	0.068	0.48 (0.27-0.88)	0.017	0.94 (0.38-2.36)	0.896
	Favorable	218	82	27	17	4,367	0.78 (0.48-1.25)	0.298	0.52 (0.26-1.03)	0.060	0.81 (0.34-1.95)	0.644

	Unfavorable	1,083	324	82	80	22,582	1 [Reference]		1 [Reference]		1 [Reference]	
High school or above	Intermediate	659	175	48	41	14,011	0.76 (0.59-0.98)	0.035	0.79 (0.44-1.42)	0.428	0.79 (0.51-1.23)	0.294
	Favorable	319	67	17	14	6,923	0.58 (0.41-0.83)	0.003	0.64 (0.38-1.10)	0.105	0.39 (0.20-0.76)	0.005
**Body mass index (kg/m2)**												
	Unfavorable	462	156	39	34	9,299	1 [Reference]		1 [Reference]		1 [Reference]	
<25	Intermediate	252	59	14	21	5,394	0.68 (0.48-0.96)	0.030	0.70 (0.28-1.72)	0.432	1.12 (0.49-2.58)	0.788
	Favorable	163	35	8	7	3,540	0.55 (0.36-0.84)	0.006	0.69 (0.34-1.39)	0.298	0.36 (0.14-0.90)	0.030
	Unfavorable	1,530	625	186	144	30,199	1 [Reference]		1 [Reference]		1 [Reference]	
≥25	Intermediate	797	294	81	59	16,097	0.76 (0.58-0.98)	0.037	0.64 (0.41-1.00)	0.049	0.82 (0.52-1.30)	0.401
	Favorable	374	114	36	24	7,750	0.67 (0.50-0.89)	0.007	0.56 (0.31-1.00)	0.049	0.55 (0.29-1.06)	0.073
**Waist circumference (cm)**												
men < 90 and women < 85	Unfavorable	387	91	20	19	8,276	1 [Reference]		1 [Reference]		1 [Reference]	
	Intermediate	232	34	6	14	5,150	0.77	0.262	0.76	0.721	1.69	0.254

							(0.48-1.22)		(0.16-3.48)		(0.69-4.14)	
	Favorable	151	23	3	7	3,348	0.51 (0.28-0.91)	0.023	0.28 (0.04-2.10)	0.215	0.75 (0.22-2.53)	0.639
men ≥ 90 and women ≥ 85	Unfavorable	1,562	668	196	155	30,476	1 [Reference]		1 [Reference]		1 [Reference]	
	Intermediate	792	308	86	66	15,875	0.73 (0.58-0.91)	0.005	0.66 (0.43-0.99)	0.045	0.80 (0.50-1.28)	0.353
	Favorable	372	119	38	24	7,704	0.64 (0.49-0.83)	0.001	0.60 (0.39-0.94)	0.027	0.49 (0.25-0.98)	0.043
**Hypertension**												
Yes	Unfavorable	826	474	151	98	14,588	1 [Reference]		1 [Reference]		1 [Reference]	
	Intermediate	427	220	69	45	8,042	0.73 (0.54-1.00)	0.050	0.69 (0.44-1.10)	0.123	0.96 (0.57-1.61)	0.875
	Favorable	202	98	33	19	3,852	0.70 (0.51-0.97)	0.031	0.74 (0.47-1.15)	0.180	0.62 (0.28-1.36)	0.231
No	Unfavorable	1,166	307	74	80	24,910	1 [Reference]		1 [Reference]		1 [Reference]	
	Intermediate	622	133	26	35	13,449	0.78 (0.61-0.99)	0.044	0.61 (0.25-1.50)	0.280	0.72 (0.39-1.31)	0.278
	Favorable	335	51	11	12	7,437	0.58 (0.40-0.84)	0.004	0.35 (0.13-1.00)	0.050	0.40 (0.16-1.01)	0.052
**Diabetes**												
Yes	Unfavorable	368	245	75	40	6,136	1 [Reference]		1 [Reference]		1 [Reference]	
	Intermediate	180	119	33	22	2,889	0.73	0.126	0.65	0.229	1.14	0.787

							(0.49-1.09)		(0.32-1.31)		(0.43-3.04)	
	Favorable	81	41	11	2	1,494	0.79 (0.45-1.39)	0.417	0.86 (0.28-2.64)	0.798	0.02 (0.00-0.12)	<0.001
	Unfavorable	1,624	536	150	138	33,361	1 [Reference]		1 [Reference]		1 [Reference]	
No	Intermediate	869	234	62	58	18,603	0.76 (0.60-0.96)	0.022	0.69 (0.40-1.20)	0.190	0.77 (0.49-1.21)	0.262
	Favorable	456	108	33	29	9,796	0.61 (0.45-0.83)	0.001	0.55 (0.33-0.92)	0.023	0.55 (0.28-1.06)	0.074
**CVD**												
	Unfavorable	191	146	62	27	2,590	1 [Reference]		1 [Reference]		1 [Reference]	
Yes	Intermediate	79	67	23	10	1,023	0.74 (0.52-1.06)	0.098	0.52 (0.29-0.92)	0.025	1.23 (0.41-3.64)	0.711
	Favorable	20	15	6	1	316	0.74 (0.29-1.88)	0.524	0.84 (0.19-3.65)	0.816	0.29 (0.03-2.64)	0.273
	Unfavorable	1,801	635	163	151	36,908	1 [Reference]		1 [Reference]		1 [Reference]	
No	Intermediate	970	286	72	70	20,468	0.74 (0.57-0.96)	0.026	0.71 (0.37-1.35)	0.297	0.81 (0.50-1.33)	0.413
	Favorable	517	134	38	30	10,973	0.63 (0.49-0.80)	<0.001	0.56 (0.33-0.94)	0.030	0.50 (0.26-0.98)	0.044
**Cancer**												
Yes	Unfavorable	120	78	18	25	1,981	1 [Reference]		1 [Reference]		1 [Reference]	
	Intermediate	50	28	6	9	884	0.68	0.182	0.48	0.199	0.55	0.288

						(0.39-1.20)		(0.15-1.48)		(0.18-1.67)	
	Favorable	27	16	7	4	439	0.74 (0.43-1.28)	0.282	1.92 (0.75-4.92)	0.173	0.38 (0.15-1.02)	0.054
No	Unfavorable	1,872	703	207	153	37,517	1 [Reference]		1 [Reference]		1 [Reference]	
	Intermediate	999	325	89	71	20,607	0.76 (0.61-0.94)	0.010	0.67 (0.42-1.07)	0.095	0.92 (0.61-1.39)	0.692
	Favorable	510	133	37	27	10,851	0.63 (0.48-0.84)	0.002	0.47 (0.30-0.75)	0.002	0.51 (0.25-1.07)	0.075
**Emphysema or chronic bronchitis**											
Yes	Unfavorable	168	96	24	25	2,843	1 [Reference]		1 [Reference]		1 [Reference]	
	Intermediate	55	26	8	4	958	0.73 (0.36-1.47)	0.377	0.39 (0.15-0.98)	0.045	(-)	(-)
	Favorable	25	8	3	0	517	0.45 (0.30-0.67)	<0.001	0.63 (0.15-2.62)	0.526	(-)	(-)
No	Unfavorable	1,824	685	201	153	36,655	1 [Reference]		1 [Reference]		1 [Reference]	
	Intermediate	994	327	87	76	20,533	0.73 (0.59-0.89)	0.003	0.62 (0.37-1.04)	0.071	0.90 (0.57-1.41)	0.643
	Favorable	512	141	41	31	10,773	0.65 (0.51-0.84)	0.001	0.57 (0.35-0.92)	0.020	0.57 (0.31-1.03)	0.064

[a] Adjusted for age, sex, ethnicity, poverty status, education level, body mass index, and prevalent comorbidities (including history of hypertension, diabetes, CVD, cancer, chronic bronchitis, or emphysema).

Table S7. Association between combinations of the healthy lifestyle factors (Prevalence >5%) and risk of liver cancer.

Healthy lifestyle score	SMK	ALCO	DIET	PA	Prevalence (%)	All-cause mortality		Cardiovascular mortality		Cancer mortality	
						Hazard Ratio (95% CI)[a]	*P* value[a]	Hazard Ratio (95% CI)[a]	*P* value[a]	Hazard Ratio (95% CI)[a]	*P* value[a]
**NAFLD**											
Score 0					753 (22.06)	1 [Reference]		1 [Reference]		1 [Reference]	
					247 (8.48)	0.82 (0.56-1.20)	0.303	0.67 (0.34-1.34)	0.258	0.99 (0.53-1.85)	0.967
Score 1					447 (12.52)	0.87 (0.67-1.14)	0.325	0.90 (0.54-1.48)	0.678	0.61 (0.37-1.00)	0.051
					491 (12.28)	0.53 (0.36-0.77)	0.001	0.58 (0.25-1.34)	0.202	0.47 (0.22-1.01)	0.052
					226 (8.21)	0.76 (0.54-1.07)	0.116	0.73 (0.30-1.80)	0.498	0.93 (0.46-1.87)	0.837
Score 2					191 (5.83)	0.43 (0.30-0.61)	<0.001	0.57 (0.35-0.95)	0.029	0.33 (0.12-0.91)	0.033
					371 (9.12)	0.52 (0.36-0.75)	<0.001	0.30 (0.17-0.55)	<0.001	0.41 (0.19-0.87)	0.021
Score 3					211 (7.47)	0.54 (0.37-0.80)	0.002	0.40 (0.18-0.90)	0.027	0.31 (0.12-0.75)	0.010
**No NAFLD**											
Score 0					1261 (21.28)	1 [Reference]		1 [Reference]		1 [Reference]	
					587 (11.06)	1.02 (0.83-1.25)	0.857	1.39 (0.87-2.21)	0.169	0.85 (0.51-1.41)	0.526
Score 1					570 (9.46)	0.81 (0.64-1.04)	0.100	0.93 (0.62-1.40)	0.738	0.54 (0.29-1.00)	0.051
					795 (11.05)	0.44 (0.31-0.62)	<0.001	0.67 (0.36-1.26)	0.214	0.30 (0.14-0.66)	0.003
					409 (8.35)	0.61 (0.47-0.78)	<0.001	0.83 (0.51-1.36)	0.462	0.29 (0.16-0.50)	<0.001
Score 2					496 (8.16)	0.64 (0.45-0.91)	0.013	0.59 (0.27-1.29)	0.188	0.37 (0.17-0.80)	0.011
					588 (8.62)	0.51 (0.38-0.69)	<0.001	0.89 (0.49-1.62)	0.708	0.21 (0.10-0.42)	<0.001
Score 3					467 (9.79)	0.56 (0.43-0.74)	<0.001	0.44 (0.26-0.75)	0.003	0.40 (0.21-0.78)	0.008

[a] Adjusted for age, sex, ethnicity, poverty status, education level, body mass index, and prevalent comorbidities (including history of hypertension, diabetes, CVD, cancer, chronic bronchitis, or emphysema).

Figure S1. Association between combinations of the healthy lifestyle factors (Proportion >5%) and risk of all-cause mortality/cardiovascular mortality/cancer mortality in the sub cohort without NAFLD. Adjusted for age, sex, ethnicity, poverty status, education level, body mass index, and prevalent comorbidities (including history of hypertension, diabetes, CVD, cancer, chronic bronchitis or emphysema).

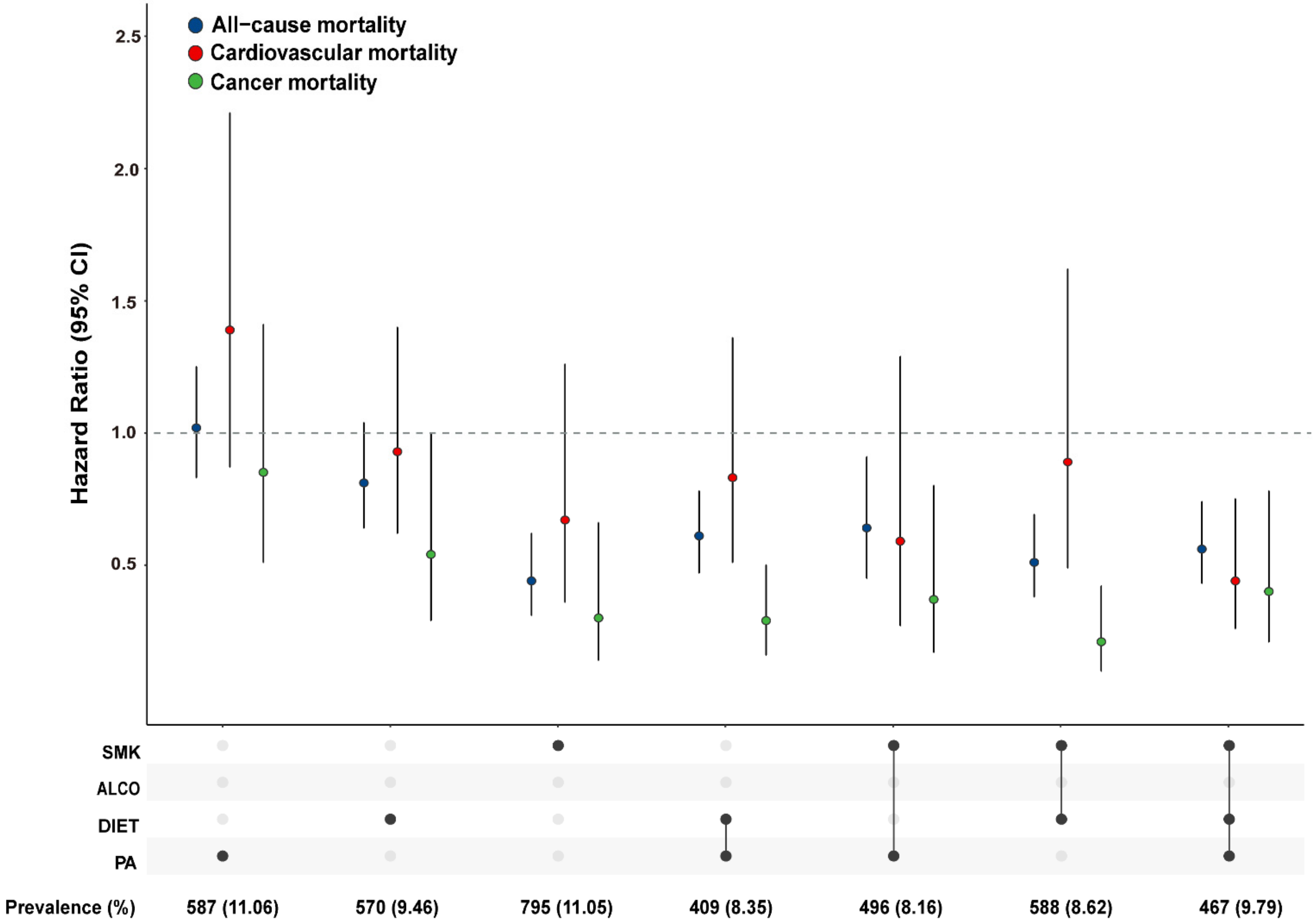

Supplement: Supplementary file 1 [file nutrients-14-03785-s001.zip › nutrients-1841923-supplementary.pdf]
